# The Machine behind the Stage: A Neurobiological Approach toward Theoretical Issues of Sensory Perception

**DOI:** 10.3389/fpsyg.2016.01357

**Published:** 2016-09-13

**Authors:** Konstantinos Moutoussis

**Affiliations:** Department of History and Philosophy of Science, National and Kapodistrian University of AthensAthens, Greece

**Keywords:** brain, perception, neuroscience, psychology, philosophy

## Abstract

The purpose of the present article is to try and give a brief, scientific perspective on several issues raised in the Philosophy of Perception literature. This perspective gives a central role to the brain mechanisms that underlie perception: a percept is something that emerges when the brain is activated in a certain way and thus all perceptual experiences (whether veridical, illusory, or hallucinatory) have a common cause behind them, namely a given brain-activation pattern. What distinguishes between different cases of perception is what has caused this activation pattern, i.e., something very separate and very different from the perceptual experience itself. It is argued that separating the perceptual event from its hypothetical content, a direct consequence of the way everyday language is structured, creates unnecessary ontological complications regarding the nature of the hypothetical ‘object’ of perception. A clear distinction between the physical properties of the real world on the one hand (e.g., wavelength reflectance), and the psychological properties of perceptual experiences on the other (e.g., color) is clearly made. Finally, although perception is a way of acquiring knowledge/information about the world, this acquisition should be considered as a cognitive process which is separate to and follows perception. Therefore, the latter should remain neutral with respect to the ‘correctness’ or ‘truth’ of the knowledge acquired.

## Neurobiology Behind Perception: Changing the Question From ‘What’ To ‘Why’

When a neuroscientist considers perception or any other mental process, the starting point is the existence of a biological ‘machine’ (Ryle, 1949), the brain, the activation of which generates all mental states and events that appear to us as if taking place on the stage of a ‘Cartesian Theater’ in the mind (Dennett, 1991). There are two main consequences arising from this thesis. The first one is the fact that the only direct cause of any mental state/event is a given pattern of brain activation: perception is created by a perceptual system, and behind each and every percept there is a certain neuronal activation, fully responsible for causing this percept. What produces the neuronal activation is a separate question: it can be physical objects sending light to the eye, artificial brain stimulation by an electrode, an epileptic seizure, magic mushrooms, auto-activation while dreaming, and many more. All these alternative brain-stimulation events can theoretically have an identical result: a specific brain-activation pattern, leading to the formation of a specific percept. The Causal Theory of Perception (see Grice, 1961; Lewis, 1980; Snowdon, 1981) is a philosophical standpoint in harmony with this view, although too much energy is wasted in trying to accurately define what veridical perception is and how it differs from illusions and hallucinations. Trying to semantically categorize different perceptual experiences into different groups can indeed be an interesting, challenging game. Regarding the nature of perception, however, it does not offer much more insight on top of the fact that specific, individual perceptual experiences are caused by specific, individual brain activation patterns. To deny that the latter is neither identical with nor constitutive of the experience itself (Child, 1994, pp. 161–162) is a step back toward dualism.

The second consequence of a brain-centered theory of perception is that, since the percept is the creation of a given neuronal system, its characteristics will depend on and directly reflect the properties of this system. This does not imply that perception is of an esoteric nature and in complete isolation from the physical world. Such isolation would miss the point, since perceptual systems have evolved in order to enable organisms to interact with their environment. In the example of vision, light falling on objects activates the brain by the process of phototransduction, during which photoreceptors at the retina transform electromagnetic energy into electrochemical activation, which in turn sends a neural signal to the rest of the visual brain. This light has specific characteristics which are determined by the properties of the reflecting object (i.e., carries information), and so determines the characteristics of the elicited brain activation. Thus, the characteristics of a percept are dictated by both the perceptual system which creates it and the properties of the physical object we are looking at. In this way we can acquire objective knowledge about the world, albeit in a very subjective manner. Perception is therefore characterized by an objective subjectivity or, to say it perhaps better, a subjective objectivity. Objectivity, since the transformation from the physical to the perceptual world follows certain constant, reliable rules. Subjectivity, since each percept is created by a perceptual system and therefore its characteristics depend on the properties of the latter: the same chair looks different to a human, a cat or a bat, and perhaps looks different even between two humans. Plato has realized that what we perceive are ‘reflexions of reality’ (Plato: The Republic, Book VII). The nature of these reflections depends on the nature of the perceptual system that both creates and perceives them.

The characteristics of the percepts created by the brain do not solely depend on the bottom-up processing of incoming sensory information, but are also determined by top-down mechanisms reflecting previous experiences of the subject. Starting from Hermann von Helmholtz more than a century ago (Helmholtz, 1866), the idea that perception could be seen as an inference process, rather than the intuitive ‘normal-picture scenario,’ has become increasing popular. A percept is the result of such inference process based on the internal representation generated by the brain. Recently, there is some further development of this line of thinking, drawn on the estimation theory and Bayesian inference from the field of statistics, to formulate mathematically rigorous models for perception that can be tested quantitatively against experimental data (for examples see Knill and Richards, 1996; Girshick et al., 2011; Clark, 2013). This shift is away from the brain as a passive filter of sensations and toward a view of the brain as a statistical organ that generates hypotheses which are tested against sensory evidence. The brain never knows anything about an object for sure but can only make maximum likelihood predictions about what an object is, how it will appear in the future, or how it will interact with different senses based on the sensory information we have at present. Perception is making predictions and thus percepts are reconstructions of the world around us that represent our best guess as to what is out there, based on the statistics of how sensory information impinges on the sense organs. In this way, the ambiguity of sensory information can be dealt with. In 3D vision, for example, the problem of reconstructing a 3D object given two 2D views is inherently under-constrained: given a homographic projection, infinitely many objects of differing size can produce the same image on the retina. It is only through heuristics and tricks based on natural image statistics that the visual system is able to generate a plausible guess at an object’s 3D structure that is correct most of the time (but not always – see ^1^ for a nice example). The reality we experience is our best guess at how to reconstruct the world based on what most probably generated our sensory inputs. In cases where the brain cannot decide on which of the explanations is most probable, we can have instances of bistable (or multistable) vision (e.g., Necker, 1832; Blake and Logothetis, 2002).

To a neuroscientist, all there is to a percept is the neurobiological mechanism behind it. This is true for veridical perceptions, illusions or hallucinations, and there is thus nothing peculiar or problematic regarding the last two. Under normal circumstances, the particular brain activation that leads to the particular percept of a chair comes around with the participation of two extra factors: a real chair and light. A whole neural process is initiated by the latter being reflected from the former, activating the retina at first, then being processed by the rest of the visual system, and at some point reaching the necessary and sufficient neuronal activation that leads to the percept. The presence of either light or a chair, however, is not necessary and definitely not sufficient. As mentioned above, the desired activation can also be achieved by alternative means – if it does, the percept is there and no real chair or light need to be there at all. Therefore, although the chair is initiating the birth of the percept under normal circumstances and although the nervous system has evolved to detect real chairs, the chair is not a part of the mental state of the subject, not even the direct cause of this mental state. In some cases it is the indirect cause, in that it causes the brain activation which in turn causes/is the mental state. Thus, instead of asking *what* it is that one perceives, it would be more informative to ask *why* one perceives something. The existence of a nervous system creating perception is always part of the answer and, quite often but not always, the presence of light and a physical object are parts of the answer too. In general, describing mental events by using language structured to relate phenomena in the physical world creates serious theoretical issues in the philosophy of perception. One of these is the separation between the verb/action, the subject acting and the object being acted upon. In my opinion, this separation is fraud and creates more problems than it solves. These problems disappear if one accepts that there is no difference between a percept and the content of that percept, i.e., no separation between the action of perceiving and a hypothetical object toward this action is directed^2^.

From the above it follows that, as far as the mental process of perceiving is concerned, there is no difference between a veridical percept, an illusion or a hallucination: all three are equivalent mental states, because they share a common brain activation pattern. Even if the activation of the brain is not identical (e.g., see Weisz et al., 2007), there is no way for the subject to know that. It is also quite likely that more than one activation-patterns lead to a particular experience, so we cannot be sure that every time we have the same experience the brain^3^ is activated in the same way. We should be more sure, however, that a particular brain activation leads to a particular perceptual experience, and thus if we are able to artificially replicate this activation we can produce a hallucination that is indistinguishable from the original, ‘veridical’ perceptual experience. People have tried to fabricate differences between them by saying that a hallucination is ‘private’ to its subject, in the sense that only the subject of the hallucination is aware of the particular experience. But this is true for stimulus-induced vision as well, since what we are aware of is not the physical object itself but rather the mental percept, which is equally private in the case of ‘veridical’ perception as in the case of hallucination. Although the knowledge that a chair is out there can be shared by many different people, each perceptual experience induced by this chair in each different nervous system is as unique and private as it comes. This issue is sometimes confused, for example by Price (1932, pp. 31–32) when he compares the perception of a real object with a hallucination and describes the latter as ‘the fleeting product of cerebral processes,’ whereas the former as ‘a real constituent of a material object, wholly independent of the observers mind and organism.’ The fact that the physical object is totally independent from the mind and the organism of the observer does not mean that the same is true for the percept that this object initiates. Without a visual system, without the existence and the activation of the relevant perceptual areas of the brain, no percept -be it veridical or hallucinatory- would exist.

## The Common Factor

A philosophical position in agreement with the brain-centered view described above is the Common Factor principle (see Fish, 2010, p. 3). This principle states that all types of perceptual experience share a common mental state – something which seems obvious to a neuroscientist, as it is a direct consequence of the presence of a common brain-activation pattern behind the common metal state. As mentioned above, it is logical to assume that activating the brain in a certain way should result in experiencing the same mental state; otherwise we would live in a world that makes little sense (apples should sometimes look red and sometimes look blue etc.). Therefore, a veridical perception and a hallucination which are phenomenologically identical, do share a common mental state as well as a common brain activation pattern. There is, however, a difference in what is causing this activation: in one case it is the presence of light and a physical stimulus, whereas in the other case the brain is activated by a different reason (see Child, 1994, p. 145 for a similar view). As far as perceptual experience is concerned, however, the two cases are identical if the same brain-activation pattern is behind both.

One could unnecessarily complicate things by saying that the two distinct mental states differ in representational content, or in their status as perceptual evidence (Martin, 1994, p. 745). However, the fact that in one case the mental state gives correct information about the world whereas in the other it gives false information, does not make them different mental states – their status as perceptual evidence depends on something external (the presence or absence of an object). Two identical veridical percepts can also be distinct, just because one takes place at 12:00 and the other at 12:01, but that doesn’t mean that they cannot share an identical mental state. Similarly, if a twin-universe exists, all mental states would come in pairs, the members of each pair being distinct identical entities. The best way to avoid such unnecessary complications is to stick to the notion that there is nothing more to a percept than its phenomenology, thus keeping representational content and belief acquisition separate from it.

The Disjunctive Theory of Perception (see Haddock and Macpherson, 2008; Byrne and Logue, 2009) rejects the Common Factor principle and states that hallucination and perception do not share a common mental state. A common example used to challenge the idea that two percepts which are indiscriminable should necessarily share the same phenomenology is the following (see Fish, 2010, p. 152): stimulus B cannot be distinguished from stimulus A because their intensity difference is below their just noticable difference (JND), as is the intensity difference between C and B (where I_A_ < I_B_ < I_C_), but yet one could distinguish between A and C if their intensity difference is above JND. The argument is that if indiscriminability implies identical phenomenology, one should not be able to distinguish between A and C either. From this it follows that veridical percepts and hallucinations could still have a different phenomenology despite being indiscriminable. However, the JND depends on the adaptation state of the system and therefore the phenomenology of B is different to the phenomenology of C for a system adapted to A, but not for a system adapted to B. If we call these two phenomenologies B1 and B2, respectively, A cannot be distinguished from B because both have the same phenomenology B1, whereas B cannot be distinguished from C because they both have the same phenomenology B2. Therefore, A and C have a different phenomenology and this is why they can be discriminated.

Although the JND argument does not demonstrate a case in which indiscriminable objects have a different phenomenology, we cannot rule out the possibility that this could theoretically happen. Nevertheless, given that the phenomenology of a perceptual experience is fully determined by the pattern of brain activation behind it, if the technology was available to bring the brain in the exact same state as a particular physical stimulus does, one would experience the exact same percept. A veridical percept and a hallucination would then have exactly the same phenomenology, as well as the same underlying mental and neural states. A negative disjunctivist would claim that the two share a common property, but the former has a special property in addition, the effects of which the latter inherits via their common property (see Fish, 2010, p. 100). However, this is just a more complicated way of saying that veridical perception is special because the brain activation is caused by light coming from a physical object. The two ‘types’ of perception continue to share a common brain-activation pattern, a common mental state and a common phenomenology. What they do not share is the presence or absence of an object, something which lies outside the perceptual experience itself but rather belongs to the reality of the physical world.

## On the Nature of the Perceptual ‘Object’

Focusing on the brain could give a simple, straightforward answer to one of the major questions in Philosophy of Perception, namely *what* is the object of perception. When I look at a chair, what is it that I really experience? Is it the chair itself, the physical object? Or is it some sort of a ‘mental object’? What is the ‘content’ of my perception, and what is the nature of this content? Such puzzles and ontological commitments disappear if one adopts the idea that a distinction between a percept and its content (see Martin, 1994, p. 464) is not necessary at all and thus there is no object, be it mental or physical, that is being perceived. Indeed, the distinction between a subject, a verb and an object that is so fundamental in everyday language, does not seem appropriate to describe the perceptual process and the mental world in general, nicely demonstrating the fact that philosophical issues are sometimes more a matter of linguistic practices than anything else.

In a first attempt to overcome the ‘special’ cases of illusion and especially hallucination, in which there is no physical object that we perceive, the theory of *Sense Data* states that (in general) we perceive mental rather than physical entities (see Robinson, 1994). This approach solves the problem of perceiving non-existing objects during illusions, hallucinations, imagery, dreaming etc. Furthermore, it takes care of the so called time lag argument, which is that it takes some time for physical stimuli to reach our eyes and get processed by our visual system, and therefore what we experience is something that could very well not exist as such in the physical world any more (Russell, 1927, p. 155; Russell, 1948, p. 204). However, Sense Data theory is problematic regarding the nature of a non-physical object: it is common to talk about *mental states* or *events*, but what exactly would such a thing as a *mental object* be? It was hinted earlier that equating perception with its content gets rid of this dilemma regarding the ‘owner’ of the features that we perceive, as well as of the necessity to create mental objects, such as sense data, possessing these properties. The *Adverbial Theory* of perception does exactly that, eliminating the distinction between perception and its object. Instead of talking about *objects* which are being sensed, it appeals to *ways* of sensing (see Jackson, 1975). For example, instead of saying ‘John saw red’ we can say ‘John saw redly,’ thus getting rid of the necessity of the existence of a red object (be it physical or mental) that is being sensed. Do such theories isolate perception from the physical world? As explained earlier, despite its phenomenological subjectivity, perception can provide us with constant, reliable, objective information about our physical environment. It doesn’t matter what a good-to-eat banana looks like to a monkey, as long as good bananas always looks this way to the particular monkey, while bad bananas look a different way. Similarly, the fact that the relationship between the symbol 38.7 and a body of high temperature is totally arbitrary does not mean that thermometers are of no use in giving us valuable information. This has been realized by Locke, who argues that perceptual experiences are subjective but reliable signs of their regular causes: ‘...the Idea of Whiteness, or Bitterness, as it is in the Mind... has all the real conformity it can, or ought to have, with Things without us’ (Essay, IV. iv. 4).

In trying to answer the question of why perceptual experiences have the phenomenology that they have, the Phenomenal Principle states that if we are aware of a quality, there must be *something* of which we are aware that possesses this quality (Price, 1932, p. 3; Robinson, 1994, p. 32). If a physical object is implied by *something*, then the principle is wrong given the existence of things such as hallucinations, dreams, epileptic seizures, artificial brain-stimulation, magic-mushrooms etc. If, on the other hand, *something* refers to a mental entity like a percept, we have the problem of artificially separating the percept from its content – not to mention the arbitrary introduction of a *self*, magically observing all this from the ‘outside’. The Adverbial theory rejects the Phenomenal principle but has still the problem of demanding a separate observer for the perceptual experience, an observer who is separate from the experience itself. However, it would be both simpler and more appropriate to say that there is only one single *something* that exists: the mental state itself. A neurobiologist would thus replace the Phenomenal Principle with a statement like this: ‘For each and every percept experienced, there exists a certain neuronal activation generating it, and there might or might not be an object in the real world, whose physical properties determine the pattern of this activation and therefore the properties of this percept.’

## So is Anything Being ‘Represented’?

The classical question of whether perception is representational or not, i.e., whether a percept has an intentional content that represents the world as being some way (Martin, 1994, p. 745; Byrne, 2001), is another good example of how some theoretical issues on perception are mainly a matter of language and definitions. The idea that a percept must necessarily be *about* something is the basic view of *representationalism* (Tye, 1995, 2009), which is in opposition to pure sense-datum theorists that do not accept visual experiences to have any intentionality. For the scientist, on the other hand, an object in the physical world sends a particular light composition to the eye of the observer, which causes neuronal activation at several levels of the visual system. The relationship between each of these activation patterns and the properties of the object is not chaotic, but follows particular algorithms and transformations of the signal that create several *neuronal representations* of these properties at different levels in the brain. Thus, in the case of stimulus-driven vision, the process of creating perception in the visual system contains several different representations of the information regarding the stimulus characteristics. The relationship between the physical stimulus and the emerging percept is also deterministic (for a specific perceptual system), from which it follows that we also have a *mental representation* of the physical properties of the object. So if the term ‘representational’ means that something has a given relationship to something else, like the body temperature to the thermometer reading, then stimulus-driven perception is clearly representational.

What happens when the brain activation is not elicited by a visual stimulus, like in a hallucination, in which case there is no representational relationship between neuronal and mental events and the characteristics of some physical object? In this case, perception is not representing something from the physical world, as would be the case with a digital thermometer having a random reading due to a failure in its electronic circuits. The fact that perception is sometimes ‘representational’ and sometimes not, is not such a big problem after all – it is just a more complicated way of saying that activation of the visual brain can sometimes be caused by factors other than a physical stimulus. Furthermore, the fact that percepts are mind-dependent does not necessarily mean that there is a problem in conceiving the existence of a ‘mind-independent’ world (Child, 1994, p. 149). Although it is theoretically possible that, as in the well-known science-fiction film The Matrix (1999) brains are plugged-in a supercomputer and we are living in a virtual reality, we have some clues to assume that there is something out there causing our sensory experiences: several different percepts can arise from the same physical object, all of them cease to exist if the object is removed, the latter can be experienced by other people as well, one can go to bed and find it in place next morning, and so on. Still, all this could be the result of a very successful virtual environment and there would be no real way for us to know.

As mentioned above, when brain activation is not created by the interaction of the nervous system with light and physical objects, perception loses its representational character. Categorizing the many different ways of activating the brain is not straightforward: hallucinations are cases in which perception is not caused by an object in the real world, but what if a real cat in front of a disturbed mind makes him see a tiger? Is this an illusion or a hallucination? If we say that veridical perceptions are caused by light falling on the retina from real objects, would retinal activation via a prosthetic camera count as veridical? What about the case of perceiving a non-existing person in front of me, not because of a drug that I took but because of a hi-tech, virtual-reality hologram? This issue has puzzled philosophers a great deal, and there are several even more peculiar hypothetical examples of perceptual states such as ‘The Brain before the Eyes’ and ‘The Light Meter’ (Lewis, 1980), or ‘Tom and Tim’ (Tye, 1982). What these examples show is that it is not easy to find a definition which accurately describes and distinguishes between different types of perception. However, apart from the challenging language game that this is, do we really need such definitions in order to understand what is going on? All that we need to know is that perception arises whenever the brain is activated in a particular way, by any reason or means.

## Physical Vs. Psychological Entities: The Example of Color

A good example in order to understand how percepts are *psychological* entities created by the brain rather than *physical* entities existing in the physical world is color. The science of color supports the view that phenomenal character is a property of the experience (Byrne, 2002, p. 9) rather than not (Tye, 2000), and its phenomenology can be nicely connected with known facts about the anatomy and physiology of the visual system. *Metamers*, for example, are stimuli with a different light composition that look exactly the same color, nicely demonstrating that color vision does not necessarily inform us about the precise properties of objects in the real world ^4^. Instead, the phenomenon is explained by the neurophysiological fact that there are three different cone types with different sensitivities across the visible spectrum. The Trichromatic Theory of Color Vision (see Blake and Sekuler, 2006, p. 246) can also explain the fact that any triplet of primary^5^ colors can give rise to the full gamut of the colors we perceive. Furthermore, the fact that a color cannot be red and green (or blue and yellow) at the same time, together with the fact that we need four (rather than three) names in order to roughly describe all the colors that we perceive, is a direct consequence of the way the cone input combines upstream from the retina to create opponent color-pairs (see Blake and Sekuler, 2006, p. 258). Finally, the fact that neuronal circuits in the brain compare lights coming from different part of the visual field is responsible for the well-known phenomena of color constancy and color induction^6^ (Land, 1977). Color vision thus nicely demonstrates that the characteristics of the visual experience are determined by the way in which the perceptual system is constructed. To say it with a philosopher’s words, ‘colors are a feature of the way we process visual information rather than a feature of the objective, mind-independent world’ (Fish, 2010, p. 145).

The realization that color is not a mind-independent object can generalize to perception as a whole. The old philosophical question of ‘looking red’ vs. ‘being red’ is non-existent for scientists, because there is only ‘looking red’: red is a psychological property, not a physical one, and therefore it can only exist as a result of the activation of a visual system. When a surface of high reflectance for long-wave light and low reflectance for the rest of the spectrum sends light to a primate retina, the retina transduces this light into neural signal and sends it to the thalamus. From there, the signal reaches the primary visual cortex, areas V2 and V4 and so on, and then, at some unknown point in time, the mental event of experiencing redness takes place (see Zeki, 1993 for an excellent review of the visual system). This private mental event is constant for each one of us, but could be different from one person to another. We all refer to this experience as ‘red,’ because we have agreed to give this name to the experience that we have when looking to a surface of such and such a reflectance, whatever this experience might be for each one of us. Thus, when talking about a psychological property PS such as color, nothing *is* PS but rather some things *feel* PS. Similarly, when talking about a physical property PH such as reflectance, nothing *feels* PH but some things *are* PH.

Failing to realize the distinction between physical and psychological properties is often a cause of confusion in the philosophical literature. For example, *color realism* (see Byrne and Hilbert, 2003) would claim that a percept is not red, that redness is not a property of this mental event but rather that the mental event is representing red, which is a property of an object in the physical world. This statement is incorrect, as red is indeed nothing more than a mental experience/state. As already mentioned, the presence of this experience might be related to the presence of a physical object that has a certain spectrum reflectance: light from that object falling on the retinae initiates a series of events in our visual system that lead to the creation of a red experience. If one wants to describe this by saying that the particular percept represents the particular reflectance properties of the object, then this is fine but it does not add much to our knowledge of what is going on. Furthermore, if one uses the word ‘red’ to refer to a physical property, namely a high reflectance for long-wave light, then again the problem is mainly a linguistic one. The important thing is to realize that, as physical objects have physical properties, similarly mental events (such as percepts) have psychological properties and that, whatever names one chooses for them, the two should not be confused.

Color is perhaps the most profound example in perplexing the physical with the psychological, but the problem is more generally present. Similar to the distinction between reflectance and color, there is also a distinction between oscillation amplitude and loudness, relative (to the background) energy and brightness, frequency and pitch etc. A percept can also have ‘sizeness,’ a psychological property related to the physical property of size. In the Müller-Lyer (1889) illusion, what is it that is different between the two lines that are perceived? It is not the length of the two lines, since length is a property of physical objects rather than of percepts, and also the two physical lines are of the same length. What is different is the ‘lenghtness’ of the two percepts, a psychological property which is related to the physical property of length. This relationship between the physical and the psychological has been exhaustively investigated in psychophysics, including mathematical formulas describing it (see Stevens, 1960). This is possible, despite the fact that ‘length’ and ‘lenghtness’ exist in different spaces – the physical vs. the mental. What is not possible is a comparison between them: we could never have a Muller-Lyer illusion with a single line and ask subjects to compare the lenthness of their percept with the length of the real line on the page (“does the line look longer than it is?”). One can only compare between similar things: either between physical properties, using real measurements, or between psychological properties, using measures of the psychological effect.

## More on the Psychological Nature of Percepts

Perhaps because vision is so dominant among our senses, it intuitively feels as if things in the physical world are exactly as they visually appear to us. It is quite difficult and counter-intuitive to digest the fact that the color of a red tomato shining under the sun does not belong to the tomato *per se*, but is rather a creation of our own perceptual system^7^. The physical property of the tomato which contributes to the creation of this color by the brain, i.e., the reflectance of the object for different wavelengths, is not colored at all. Therefore, it is wrong to claim that the properties of our experience belong to the object rather than to the percept. After all, nothing *feels* like anything unless there is a perceptual system there to feel it. The fallacy is more easily revealed in the case of senses other than vision. Is sweetness the property of a cake? Would a cake be sweet if there was no one to taste it? Couldn’t the same cake taste totally not-sweet to a creature having a different nervous system from the one that we have? Wouldn’t this same cake taste less sweet to the same person, if it is eaten after eating honey or while having the flu (Locke, 1961, p. 124)? Sweetness is as much a property of the cake as color is a property of the tomato. One can still argue that a representation is taking place here, as long as it is clearly understood that there is nothing ‘sweet’ in the properties which are being represented (i.e., the chemical composition of the cake). The latter are neither sweet nor sour, the only property they have is the *ability* to activate the (particular type of) brain in a way that generates the experience of sweetness. The exact same arguments apply equally well in vision, but the reflexive intuition against them is much stronger in this case.

Hearing sounds is another good example. When a dog barks, is what we hear a property of the dog? Or is it a property of its larynx? Perhaps it could be a property of the air? Or of some other physical object? What is being represented in such a case – what is the content of the representation? When a dog barks, the sound that we are experiencing is a property of our percept or, more correctly, it is the percept itself, created by our nervous system with the help of a dog moving its vocal chords in a medium full of air at atmospheric pressure. Enquiring into the intentional character of perception, we can have several representations here depending on the flexibility of defining the term ‘represents’: one could say that the percept represents the movement of the dog’s vocal chords, or equally well say that it represents the dog’s mood. As mentioned earlier, it would be simpler to abandon such unnecessary complications regarding ‘representations,’ ‘intentionality,’ ‘content’ etc., and just ask about what is *causing* the perceptual experience. If the answer includes objects/events from the physical world, then this is proof that the perceptual world does not exist in a vacuum. In the example of the dog, we perceive the barking because there is a thief there, that the dog sees, something which excites the dog, which makes it move its vocal cords, which produces pressure changes in the surrounding air, which excites cells in our cochlea and eventually leads to the activation of auditory cortex and other parts of our brain.

The fact that what we perceive are subjective properties of our percepts rather than objective properties of physical objects is also evident in cases in which the information entering the system is open to more than one interpretations by the brain. The Necker cube (Necker, 1832) is a classic example, and so are other forms of bistable perception, such as binocular rivalry (Blake and Logothetis, 2002). When a spinning dancer appears to change direction in a stochastic manner without any change in the physical stimulus itself ^8^, which property of the latter is being ‘represented’ by perception? Moving objects in the physical world can also change their direction continuously, but at any particular point in time they *have* a particular direction (not true in the case of bistable motion). An intentionalist would perhaps argue that bistable motion is the represented stimulus property, but this is not very satisfactory because such a property does not seem to belong to the physical world as we understand it. It would be better to just say that the properties of the physical stimulus activate the brain in a way that produces stochastic alternations between two alternative percepts. The phenomenology of our experience thus belongs to the experience itself, and there is no reason to believe that things are different in normal cases of motion perception, in which the physical stimulus is indeed moving in a certain direction at any given time. What we experience in both cases is a psychological property of the corresponding percept, not a physical property of the worldly object. Similarly, we can have cases in which motion is perceived despite the stimuli being static, such as the motion-aftereffect (Wohlgemuth, 1911).

## Is There a ‘Correct’ Sensing of the Physical World?

The so-called *transparency-claim* states that when we introspect our experiences, what we perceive are not features of the experience but rather features of the worldly objects (Harman, 1990; Tye, 2000). This sound like a good thing, because it means that we are able to experience the world *as it is* rather than *as we see it*. Unfortunately, it should be clear by now that when one introspects his own perceptual experience, the only thing he finds there are the properties of the experience itself. If the transparency principle was true and we could ‘see through’ our percepts to the real world directly, how could we tell the difference between seeing an object and touching an object? It could perhaps be argued that an object has two types of properties, those to be seen and those to be touched, but this is just a more complicated way of saying that the object has properties that can activate either the visual or the somatosensory system. Along this line of thought, Crane attacks representationalism by saying that when one removes his spectacles making objects appear blurry; this blurriness is clearly a property of the experience rather than of the object (Crane, 2003). However, his example silently assumes that with the spectacles on we perceive the world *as it is*, leaving the door open to counterarguments such as that we are actually not aware of the blurriness but simply fail to represent the sharp boundaries of the object (Tye, 2003). The truth is, spectacles or no spectacles, what we perceive (sharpness, blurriness, color, motion) is the result of the interaction of the physical stimulus with our visual system. The former includes the physical object, light, the medium, spectacles – everything that is outside our visual system. The latter will transform, in an unknown but predetermined and reliable way, physical properties to psychological properties, which constitute our visual experience.

The usefulness of perception derives from the fact that its characteristics do not only depend on the properties of the perceptual system creating it, but also on the characteristics of the physical objects in the real world. In Plato’s allegory, the shape of the shadows seen by the chained prisoners depends on the shape of the things passing by behind them, in front of a burning fire. Objectivity is further assured by the fact that there is a given deterministic way, a constant ‘algorithm’ which transforms the physical stimulus into neuronal activation and thus to the emergence of a specific percept. For a given perceptual system, a red apple will always look red and a yellow banana will always look yellow, irrespective of what ‘red’ and ‘yellow’ is like for that particular system. Perception can ‘...yield knowledge of an objective world beyond experience, and... put us in a position to think about such a world...’ (Child, 1994, p. 148). In this way our perceptual experiences make sense, helping us to detect constant properties of our environment and gain knowledge about the world in which we exist, thus satisfying the ‘epistemological hat’ of philosophical enquiries (Fish, 2010).

Since percepts are subjective by virtue of the fact that they are created by perceptual systems, could this mean that perception hides from us the ‘truth’ of the real world? ‘If all I ever get is smoke, how do I know what fire is like?’ (Campbell, 2002, p. 6). The problem with this way of thinking is that only a percept *is* (experientially) like something – the question has no meaning with respect to a physical object. One can complain that the percept of smoke is different to the percept of fire, but it is false to wonder whether a physical object in reality feels different from the experience that it produces. For example, Butchvarov (1980, p. 273) claims that having a headache because of the presence of carbon monoxide does not mean that you are conscious of carbon monoxide. This is wrong: you *are* conscious of the presence of CO by virtue of having a headache – having the perceptual experience of a headache can equally well amount to being conscious of the presence CO as when (hypothetically) seeing it, touching it, smelling it, hearing it. You can also have a headache from other reasons as well, say from CO_2_, and if the two types of headaches feel different, then this is a good way to separate being conscious of the presence of CO versus being conscious of the presence of CO_2_. Even if they feel the same it is not a problem – as mentioned above, in vision there are numerous examples of different stimuli leading to the identical percept.

Most scientists would probably agree with *naïve realism* on the fact that the external world shapes the contours of conscious experiences (Martin, 2004, p. 64), but would disagree on the view that the objects of awareness are actually the mind independent objects that inhabit the world (Fish, 2010, p. 96). The idea that we perceive the world ‘directly’ or ‘as it is’ can be disputed by the fact that what reaches our brains is nothing more than a neuronal signal, the result of sensory transduction at the sensory receptors. Therefore, no light, or pressure, or objects, or anything that exists in the physical world can enter into our neuronal and mental universe. The physical world does not ‘look’ or ‘feel’ like anything, unless there is a perceptual system to look at it or feel it. It follows that our subjective experience of the world is neither correct nor wrong, as the latter does not have a ‘proper’ or ‘true’ experiential quality on its own (unlike the case in Plato’s cave, where objects have true appearances). The definite answer to Berkeley (1910) is that if a tree falls in a forest and there is no one there to hear it, there will be no sound. Similarly, the forest will not look, smell or feel like anything, unless there is a perceptual system there to create the corresponding perceptual experiences. So, to the question of whether we experience the physical world ‘correctly’ (Crane, 1992, p. 139), the answer a scientist would give is that we do not experience the world neither *as it is* nor *as it is not* (Fish, 2010, p. 3), since without a perceptual system the world alone has no perceptual quality.

## Conclusion

A percept is something that emerges when the brain is activated in a certain way and thus all perceptual experiences (whether veridical, illusory, hallusinatory etc.) have a common cause behind them: a given brain activation pattern. What distinguishes between different cases of perception is what has *caused* this activation pattern: light coming from a real object, an epileptic seizure, chemicals, artificial stimulation with electrodes implanted in the brain and so on. All of these cases and many others, however, have a factor in common: what causes the emergence of the percept is the fact that the brain is activated in a particular way. Such a brain-centered view of perception satisfies both the phenomenological and the epistemological ‘hat’ (Fish, 2010) in a pretty adequate manner: the former because it explains the properties of our perceptual experiences as emerging from the properties of our perceptual systems, and the latter because it grounds these experiences to the properties of the objects in the physical world. Therefore, we do not need to appeal to the presence of mental objects, as sense data theory does, or qualia (Crane, 2000), as the adverbial theory does, in order to explain why perceptual experiences feel the way they do. We also do not need to wonder about whether we perceive the world as it is, because there is no such thing as a ‘correct’ perception: the physical world does not feel like anything, unless there is a sensory system to create a perceptual experience of it. Finally, we need not worry that perception is perhaps useless, since our brains can, via their sensory organs, interact with the physical world and produce activations (and thus mental states) that are determined by the properties of physical objects, making our perceptual experience ‘connected’ to the reality of our environment and able to provide us with information about the latter.

## Author Contributions

The author confirms being the sole contributor of this work and approved it for publication.

## Conflict of Interest Statement

The author declares that the research was conducted in the absence of any commercial or financial relationships that could be construed as a potential conflict of interest.

## Footnotes

1 https://www.youtube.com/watch?v=A4QcyW-qTUg

2 Furthermore, there is no separate subject to whom all these perceptual events are displayed – the subject is nothing more but the collection of these and other mental states and events.

3 The fact that the two events take place at different times and that the brain is not a fixed object, means that we are probably also talking about two different brains. Furthermore, since noise plays a central role in neuronal responses, even the ‘same’ brain is never activated in exactly the same way.

4 Freeman and Simoncelli (2011) have shown that grossly distorted visual images can also be metameric in peripheral vision – another case in which different physical objects can lead to a common percept.

5 In such a triplet, none of the three colors can be synthesized from a combination of the other two.

6 Colour constancy refers to the fact that colors do not change much with changes in illumination, whereas color induction to the fact that we can change the color of a surface by changing the color of its surround.

7 However, back in Isaac Newton (1704) wrote: ‘For the rays to speak properly are not colored. In them there is nothing else than a certain power and disposition to stir up a sensation of this or that color.’

8 https://en.wikipedia.org/wiki/Spinning_Dancer
